# Dielectric properties of porous silicon for use as a substrate for the on-chip integration of millimeter-wave devices in the frequency range 140 to 210 GHz

**DOI:** 10.1186/1556-276X-9-418

**Published:** 2014-08-21

**Authors:** Panagiotis Sarafis, Androula Galiouna Nassiopoulou

**Affiliations:** 1 NCSR Demokritos, INN, Terma Patriarchou Grigoriou, Aghia Paraskevi, 15310 Athens, Greece

**Keywords:** Porous Si, Nanoscale semiconductors, RF characterization, Dielectric permittivity, Millimeter-wave passive devices

## Abstract

In this work, the dielectric properties of porous Si for its use as a local substrate material for the integration on the Si wafer of millimeter-wave devices were investigated in the frequency range 140 to 210 GHz. Broadband electrical characterization of coplanar waveguide transmission lines (CPW TLines), formed on the porous Si layer, was used in this respect. It was shown that the dielectric parameters of porous Si (dielectric permittivity and loss tangent) in the above frequency range have values similar to those obtained at lower frequencies (1 to 40 GHz). More specifically, for the samples used, the obtained values were approximately 3.12 ± 0.05 and 0.023 ± 0.005, respectively. Finally, a comparison was made between the performance of the CPW TLines on a 150-μm-thick porous Si layer and on three other radiofrequency (RF) substrates, namely, on trap-rich high-resistivity Si (trap-rich HR Si), on a standard complementary metal-oxide-semiconductor (CMOS) Si wafer (p-type, resistivity 1 to 10 Ω.cm) and on quartz.

**PACS:**

84.40.-x; 77.22.Ch; 81.05.Rm

## Background

The co-integration of radiofrequency (RF) and millimeter-wave passive devices with complementary metal-oxide-semiconductor (CMOS) circuitry is quite challenging due to the low resistivity of the CMOS Si substrate that introduces important losses during electromagnetic wave propagation (eddy currents into the substrate). Another drawback towards this integration is the high permittivity of Si (*ε*_*r,Si*_ = 11.7) that causes an increase in crosstalk between lines, a decrease in antenna efficiency, and a reduction of the frequency of operation of the inductors. A viable solution recently investigated towards this integration is the formation of a local substrate with the appropriate dielectric properties on the Si wafer, on which the RF and millimeter-wave devices will be integrated. Such a substrate is a thick porous Si layer with high porosity, which can be optimized for best device performance by choosing the appropriate layer thickness, in order to minimize electromagnetic propagation losses into Si, and the appropriate low values of the dielectric permittivity, *ε*_*r*_, and loss tangent. These last values are tunable by changing the material structure and morphology [1-6].

Porous Si structure (pore size, inter-pore distance) and morphology affect all its macroscopic properties (electrical, mechanical, optical, etc.) [7]. An intensive effort was made in the literature to correlate the electrical properties of the material with its structural parameters [8-12]. In view of the application of porous Si for the on-chip integration of RF and millimeter-wave devices, its dielectric properties (dielectric permittivity and loss tangent) as a function of frequency should be known, in order to be used by the device designer for an accurate prediction of device operation. In addition, since the dielectric properties of the material depend strongly on its structure and morphology [13], it is desirable to have an experimental method to extract the dielectric parameters of the specific material used in each application.

In this work, we will first discuss the existing models that correlate the structural properties of porous Si (porosity and morphology) with its dielectric properties and we will compare them with results obtained by a broadband extraction method, based on the measurement of the S-parameters of coplanar waveguide transmission lines (CPW TLines) integrated on the porous Si substrate. By combining these measurements with electromagnetic simulations, the dielectric permittivity and loss tangent of the substrate (porous Si) can be obtained. This method has been previously used by the authors to extract the dielectric parameters of porous Si in the frequency range 1 to 40 GHz [13,14]. In this work, measurements are extended to the frequency range 140 to 210 GHz. Finally, by comparing the performance of CPW TLines on porous Si and three other substrates used in RF, namely, a trap-rich high-resistivity (HR) Si substrate [15-17], a standard CMOS Si wafer (p-type, resistivity 1 to 10 Ω.cm), and a quartz substrate, we demonstrate the superiority of porous Si as a local substrate for RF and millimeter-wave on-chip device integration.

## Methods

### Fabrication of CPW TLines on porous Si layers

**Figure 1:**
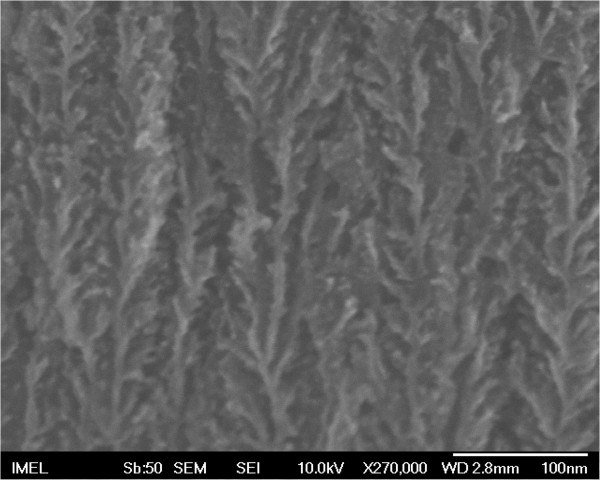
**SEM image of highly porous Si.** SEM image of highly porous Si formed on p ^+^ Si with resistivity 1 to 5 mΩ.cm. It depicts the vertical pores with dendrite structure of the material. Pore size is between 9 and 12 nm.

**Figure 2:**
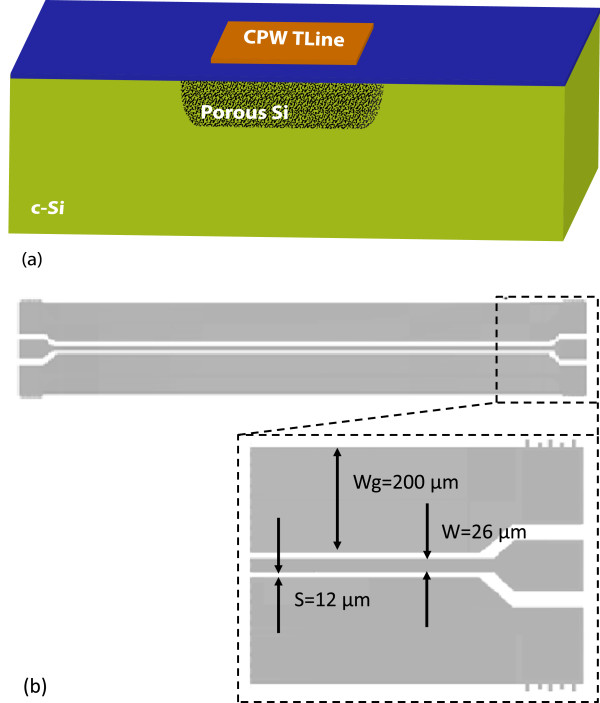
**Schematic representation of local porous Si layer on Si wafer and geometry of CPW TLine. (a)** Schematic representation of the locally formed porous Si layer on the Si wafer, on which the CPW TLine is integrated. **(b)** Topology of the CPW TLine with respective dimensions.

Thick PSi layers (thickness ~150 μm) were formed by anodization of highly doped p-type Si (*ρ* = 1 to 5 mΩ.cm). The electrolytic solution was a mixture of HF and ethanol (3 EtOH(99.9%)/2 HF(50%) v.v.) and the anodization current density was *J* = 20 mA/cm^2^. The resulting layer had a porosity of 76% and a dendritic structure as presented in Figure 1. The porous Si layer was capped with 500 nm SiO_2_ in order to stabilize it over time and achieve better planarization of the porous Si surface for further processing. On top of PSi, covered by SiO_2_, a set of coplanar waveguide transmission lines (CPW TLines), made of 1-μm-thick patterned Al, was integrated (see Figure 2).

For comparison, identical CPW TLines were also fabricated on three other substrates, as follows: the first was the state-of-the-art trap-rich high-resistivity (HR) Si RF substrate [15]. This substrate was an n-type HR-Si wafer with nominal resistivity higher than 10 kΩ.cm, covered by a bilayer of a 500-nm-thick trap-rich poly-Si layer, deposited by low-pressure chemical vapor deposition (LPCVD) at 625°C, and a-500 nm-thick TEOS SiO_2_ layer. The trap-rich layer is used to minimize the parasitic surface conduction within the Si layer underneath the silicon oxide by trapping the parasitic charges and thus restoring the initial high resistivity of the Si substrate [17]. The second substrate was a 380-μm-thick standard Si wafer used in CMOS-integrated circuits (ICs) (p-type, resistivity 1 to 10 Ω.cm). Finally, the last substrate was a 500-μm-thick quartz substrate, which is one of the off-chip RF substrates with almost negligible losses. This last substrate was used for comparison with the three other Si-based substrates.

### RF measurements and de-embedding

The S-parameters of the CPW TLines were measured in the 140-to-210-GHz range with an HP 8510B vector network analyzer (VNA) from Agilent (Santa Clara, CA, USA), combined with a millimeter-wave VNA extension module by Oleson Microwave Labs (Morgan Hill, CA, USA). All the measurements were calibrated using the Line-Reflect-Reflect-Match (LRRM) algorithm of the WinCal software from Cascade Microtech (Beaverton, OR, USA). A de-embedding procedure is always necessary in order to decouple the device response from the parasitics due to the contacts and pads. The method followed was the two-line method, using the measured S-parameters of two lines with different length (8 mm and 500 μm) [18]. The characteristic impedance, the propagation constant, and the effective permittivity of the lines were extracted and used to derive the dielectric parameters of the PSi substrate [13,14].

**Figure 3:**
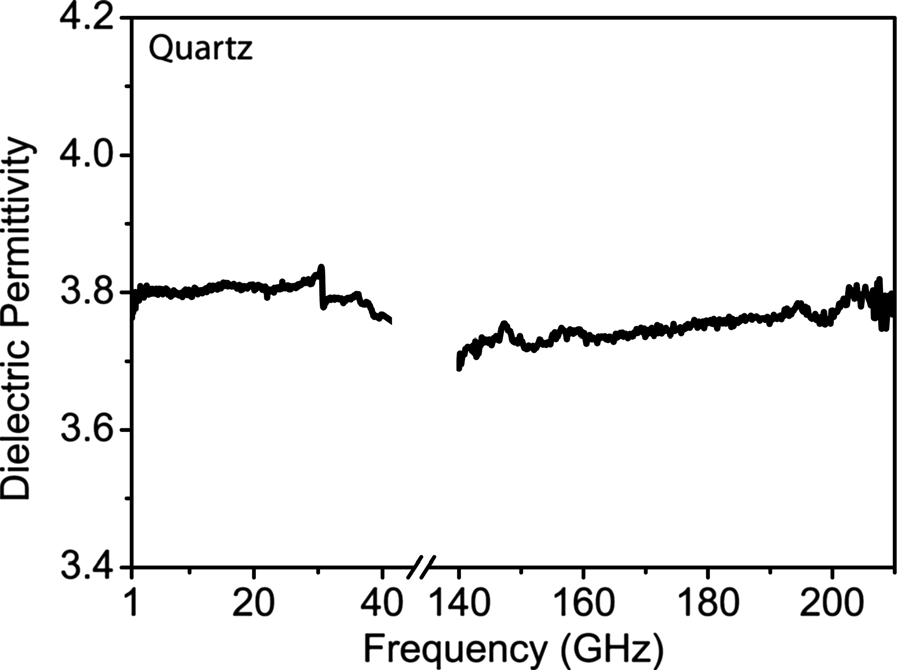
**Dielectric permittivity of quartz as a function of frequency in frequency ranges 1 to 40 GHz and 140 to 210 GHz.** The extracted dielectric permittivity of quartz as a function of frequency using the extraction method described in the text is depicted. A constant value of approximately 3.8 is obtained for the frequency range 1 to 40 GHz and on average 3.76 for the frequency range 140 to 210 GHz. The obtained values are very close to the nominal value of quartz permittivity in the whole frequency range under discussion (3.82).

The de-embedding and the extraction method were first tested for the quartz substrate (fused silica), which is known to have a constant dielectric permittivity of 3.82 throughout the whole frequency range 1 to 210 GHz [19,20]. The extraction method is described in detail in [13]. The obtained results are depicted in Figure 3 for the frequency ranges 1 to 40 GHz and 140 to 210 GHz. We can see that the curves show continuity between the two frequency ranges and the extracted values of the permittivity are 3.82 for frequencies in the range 1 to 40 GHz and 3.71 to 3.79 for frequencies in the range 140 to 210 GHz. These results are very close to the literature value of quartz permittivity (3.82) and give confidence that the de-embedding and the parameter extraction methods are valid. They were thus used to characterize the porous Si layer in the above frequency ranges.

### Microscopic models for determining PSi dielectric properties

Porous Si structure and morphology depend on the electrochemical conditions used for its formation as well as on the starting wafer resistivity. Its dielectric properties are highly dependent on its structure and morphology. There are several works in the literature that correlate the material structure with its dielectric properties. According to [9,21,22], the ac electrical transport of porous Si follows two mechanisms. The first is limited by the length of the carrier random walk through the fractal structure of the material and is valid in the very low frequency range, while at higher frequencies, the random path is shorter and the hopping length stops to be the critical factor. In that case, conduction is mainly determined by the distance between inhomogeneous areas [22]. The dielectric permittivity of porous Si (*ε*_*PSi*_) describes the polarization of the atoms and the impurities inside the material. As it is shown in [22], *ε*_*PSi*_ depends on frequency only for frequencies <100 Hz. For higher frequencies, its value is saturated and remains constant up to at least 100 kHz. This value is also independent of temperature.

### Effective medium approximation

Instead of considering carrier transport at the nanoscale, it is easier to correlate the dielectric properties of the material with macroscopic parameters, as for example the material porosity. An effective medium approximation (EMA) is used in this respect. This approximation is valid when the wavelength of the signal is much larger than the typical dimensions of pores and nanostructures composing the material. The most common models used in the literature to correlate the permittivity of non-oxidized porous Si with its porosity are the following:

*Vegard’s approximation*[1]

Vegard’s approximation is a simple mixing model correlating the dielectric permittivity with porosity (P) through the relation:

(1) $$\varepsilon_{\text{PSi}}=P\varepsilon_{\text{air}}+\left(1-P\right)\varepsilon_{\text{Si}},$$

where *ε*_*PSi*_ is the permittivity of porous Si, *ε*_*air*_ is the permittivity of air, and *ε*_*Si*_ is the permittivity of Si and P is the porosity.

*Maxwell-Garnett’s approximation*[23]

This is valid for systems in which the filling fraction *f* (where *f* = 1 - *P*) of the porous material is far smaller than the porosity (*P*) [23]. The following expression is obtained:

(2) $$\frac{\varepsilon_{\text{PSi}}-\varepsilon_{\text{air}}}{\varepsilon_{\text{PSi}}+2\varepsilon_{\text{air}}}=\left(1-P\right)\frac{\varepsilon_{\text{Si}}-\varepsilon_{\text{air}}}{\varepsilon_{\text{Si}}+2\varepsilon_{\text{air}}}$$

*Bruggeman’s approximation*[23].

This is applied to structures where the filling fraction is comparable to the porosity [23]. The following expression relates the dielectric permittivity with the porosity:

(3) $$P\frac{\varepsilon_{\text{PSi}}-\varepsilon_{\text{air}}}{\varepsilon_{\text{PSi}}+2\varepsilon_{\text{air}}}=\left(1-P\right)\frac{\varepsilon_{\text{Si}}-\varepsilon_{\text{air}}}{\varepsilon_{\text{Si}}+2\varepsilon_{\text{air}}}$$

*Bergman’s approximation*[24]

It introduces the spectral density function *g(n,P)* to take into account the nanotopology of the material. The following expression is obtained:

(4) $$\varepsilon_{\text{PSi}}=\varepsilon_{\text{air}}\left(1-f\overset{1}{\underset{0}{\int }}\frac{g\left(n,f\right)}{\frac{\varepsilon_{\text{air}}}{\varepsilon_{\text{air}}-\varepsilon_{\text{Si}}}-n}\text{dn}\right)$$

From all the above models, Vegard’s approximation is the simplest one. The most commonly used model is the Bruggeman’s model [11,25]. Both the Vegard’s model for non-oxidized Si and the Maxwell-Garnett’s model have been proven to be insufficient to explain the results of several experiments [13,24,26]. An improved version of the Vegard’s model incorporates also the SiO_2_ native oxide surrounding the Si nanostructures composing the material [27]. Better agreement between the model and experimental results is obtained in this case. The oxidation of the Si skeleton leads to a decreased permittivity of the material [11,27]. This is because the oxidation not only changes material composition, but also leads to reduction of material porosity. Finally, the Bergman’s approximation predicts quite well the dielectric behavior of PSi in the optical frequencies. The spectral density function *g(n,f)* that describes the micro-topology of the material has to be extracted in this respect [12].

### Dielectric parameter extraction using broadband electrical measurements

The models of Vegard, Maxwell-Garnett, and Bruggeman, as presented above, relate *ε*_*PSi*_ with material porosity. However, they were insufficient to explain the experimental results of several groups [13,26,28]. This can be attributed to the complexity of the PSi structure and morphology, which differs from one sample to another, even if the macroscopic porosity is the same. It is also quite difficult to find a representative *g(n,f)* function that accurately describes the specific porous Si structure and morphology in each case, making the Bergman’s model difficult to use. It is thus interesting to have a method to characterize the specific PSi material used in each experiment by simply measuring the electromagnetic (EM) response of a device integrated on it. This is the approach we use in this work. By integrating CPW TLines on top of porous Si and measuring their S-parameters, we extract porous Si dielectric parameters by combining the experimental results with electromagnetic simulations and conformal mapping calculations. This method has been described in detail in [13,14], and the results have been proven to be in very good agreement with full-wave EM simulations [14].

**Figure 4:**
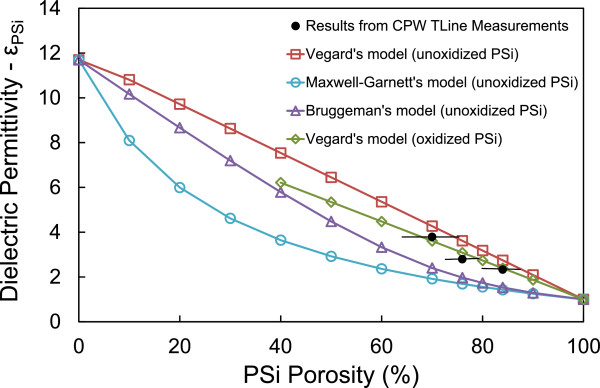
**Dielectric permittivity of porous Si as a function of porosity.** Full black dots: extracted values of the dielectric permittivity *ε*_*PSi*_ of porous Si from measurements of CPW TLines. Open squares: results using Vegard’s model for unoxidized porous Si. Open circles: results using Maxwell-Garnett’s model for unoxidized porous Si. Open triangles: results using Bruggeman’s model for unoxidized Si. Open rhombi: results using Vegard’s model for oxidized porous Si.

In Figure 4 the extracted dielectric permittivity of three PSi layers with 70%, 76%, and 84% porosity using the above method are depicted in full black circles. The PSi layers were fabricated on a p^+^-type Si wafer with resistivity 1 to 5 mΩ.cm and had a surface area of 4 cm^2^. Identical transmission lines were integrated on all three samples (see Figure 2b). The obtained results were compared with those obtained using Vegard’s, Maxwell-Garnett’s and Bruggeman’s models for PSi by applying formulas (1) to (3) given above. From Figure 4, it can be seen that the values of the extracted permittivity using broadband electrical measurements of the specific CPW TLines are between those obtained with the Bruggeman’s and Vegard’s models for non-oxidized PSi. On the other hand, by using the more elaborated Vegard’s law described in [27], which takes into account the presence of a native oxide shell surrounding the Si nanostructures (in our case, we considered a native oxide thickness of 1.5 nm and a Si skeleton thickness of 10 nm), better agreement is achieved between our experimental results and the calculated ones.

## Results and discussion

### Porous Si dielectric parameters in the frequency range 140 to 210 GHz

**Figure 5:**
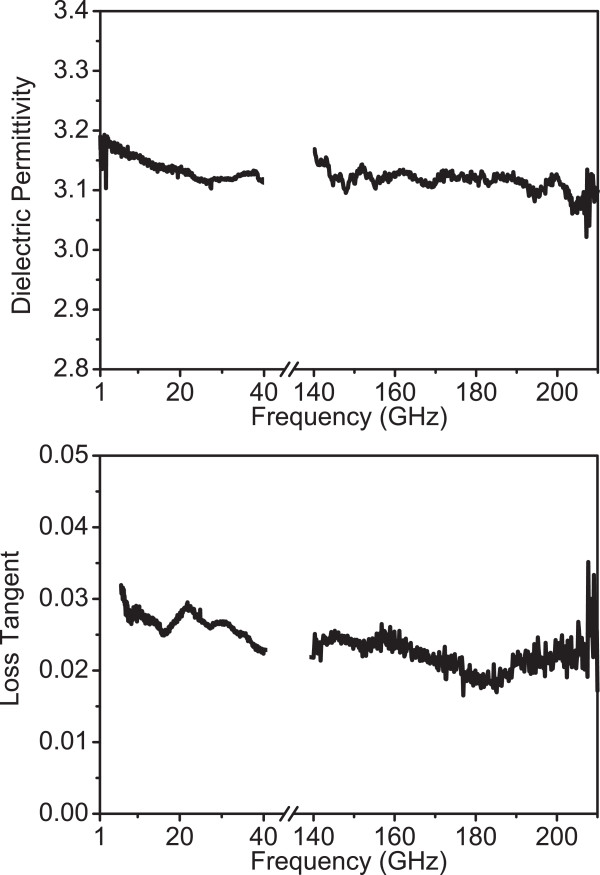
**PSi dielectric permittivity and loss tangent in frequency ranges 1 to 40 GHz and 140 to 210 GHz.** The curves depict PSi dielectric permittivity **(a)** and loss tangent **(b)**, extracted from broadband electrical measurements combined with simulations of CPW TLines integrated on the PSi substrate for the frequency ranges 1 to 40 GHz and 140 to 200 GHz.

Using broadband electrical measurements combined with simulations, the dielectric parameters of PSi in the frequency range 140 to 210 GHz were extracted. The obtained results are presented in Figure 5 in comparison with the extracted parameters for the frequency range 1 to 40 GHz. At low frequencies (1 to 40 GHz), there is an initial slight monotonic decrease of *ε*_*PSi*_ from 3.19 to 3.12 and it then stabilizes around this value (Figure 5a). In the high-frequency range (140 to 210 GHz), *ε*_*PSi*_ oscillates around the values of 3.1 and 3.2, within a maximum deviation of 0.1. Similarly, the value of the loss tangent is between 0.031 and 0.023 in the range 5 to 40 GHz (see Figure 5b), while it stays constant at 0.023 in the range 140 to 210 GHz, with a maximum deviation of 0.005. In the 1-to-5-GHz range, the results are not reliable due to the contact resistance between the RF probes and the pads and were omitted. This effect becomes negligible at higher frequencies.

In overall, from the above, we can deduce that the dielectric permittivity of porous Si is almost constant in the studied frequency ranges. It also shows a continuity of the two curves, suggesting the same constant value in the frequency range 40 to 140 GHz. The loss tangent shows a slight decrease with frequency, while again there is continuity between the low- and high-frequency curves.

### Comparison of PSi with other RF and millimeter-wave substrates

**Figure 6:**
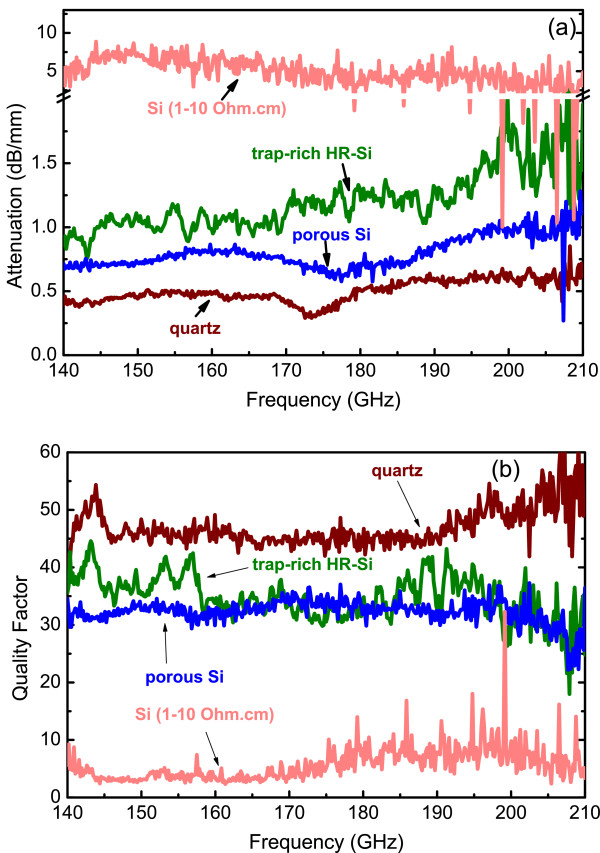
**Attenuation (a) and quality factor (b) of CPW TLines on PSi compared with three other substrates.** Comparison of signal attenuation and quality factor of CPW TLines on PSi (blue lines) compared to that of similar CPW TLines on trap-rich HR Si (green lines), quartz (dark red lines) and low-resistivity CMOS Si (orange lines) in the frequency range 140 to 210 GHz.

In order to demonstrate the high performance of porous Si for use as a substrate for RF and millimeter-wave devices, a comparison was made between this substrate and three other substrates used in the same respect. Identical CPW TLines were integrated on the four different substrates, their S-parameters were measured, and the propagation constant for each line was extracted. Figure 6 shows the extracted values of signal attenuation (a) and quality factor (b) for the CPW TLines on the four different substrates. We deduce that the lines on the three substrates, trap-rich HR Si, PSi, and quartz, have better performance than those on the low-resistivity CMOS Si. More specifically, trap-rich HR-Si reduces losses from 4.8 to 1.6 dB/mm at 210 GHz, while PSi leads to a further decrease of the attenuation loss of 1.2 dB/mm at 210 GHz. Both the above substrates show similar performance with quartz, which is a non-Si, off-chip substrate.

The observed reduction of signal attenuation *a* and the increase of the quality factor *Q* of the CPW TLine on PSi versus bulk Si is attributed to the reduction of the material loss tangent and dielectric permittivity through nanostructuring. As shown previously by the authors, the achieved low permittivity of porous Si at high porosities shows advantages in many RF and millimeter-wave devices, namely, high-characteristic impedance of the CPW TLines [5], inductors operating at higher frequencies [29,30] and antennas with reduced surface waves induced into the substrate can be obtained.

## Conclusions

The dielectric properties of a 150-μm-thick PSi layer with 76% porosity were presented for the first time for the frequency range 140 to 210 GHz, using broadband electrical measurements combined with electromagnetic simulations. It was found that the dielectric permittivity is almost constant in the above frequency range, having approximately the same value as at lower frequencies. The loss tangent is also almost constant with frequency. Finally, a comparison between the performance of CPW TLines on PSi, trap-rich HR Si, quartz, and standard low-resistivity CMOS Si was made in the above frequency range. An almost equal performance was obtained between the trap-rich HR Si, PSi, and quartz. At 210 GHz, porous Si showed an attenuation as low as 1 dB/mm and the quality factor was ~30. This performance is added to the other advantages of PSi compared to other Si-based substrates, e.g., its compatibility with the low-resistivity CMOS substrate (permitting co-integration of CMOS logic with RF and millimeter-wave devices on the same substrate) and its low achievable permittivity). All the above make PSi an excellent local substrate on the Si wafer for RF and millimeter-wave device integration on the Si chip, paving the way towards the digital/RF analog system-on-chip (SoC) of the future.

## Competing interests

The authors declare that they have no competing interests.

## Authors’ contributions

PS made the experiments and drafted the paper, while AGN supervised the work and revised the paper. Both authors read and approved the final manuscript.
